# Benzo[*a*][1,4]benzothia­zino[3,2-*c*]phenothia­zine

**DOI:** 10.1107/S2414314624003572

**Published:** 2024-04-30

**Authors:** Mamoun M. Bader, Phuong-Truc T. Pham, Salma S. Abu Khodair, Maysoon I. Saleh

**Affiliations:** a Department of Chemistry, Alfaisal, University, Riyadh 15333, Saudi Arabia; b Penn State Scranton, Dunmore, Pennsylvania 18512, USA; c Alfasial University, Riyadh, Saudi Arabia; d Department of Chemistry, The University of Jordan, Amman, Jordan; Harvard University, USA

**Keywords:** crystal structure, ladder oligomers, fused heterocyclic aromatics, dyes, donor/acceptor, organic semiconductors

## Abstract

The title compound, C_22_H_12_N_2_S_2_, crystallizes in space group *P*2_1_/*c* with four mol­ecules in the asymmetric unit. The heterocyclic mol­ecule is quasi-planar with a dihedral angle between the phenyl rings on the periphery of the mol­ecule of 1.73 (19)°. Short H⋯S (2.92 Å) and C—H⋯π [2.836 (3) Å] contacts are observed in the crystal with shorted *π*–π stacking distances of 3.438 (3) Å along the *b* axis. Surprisingly, and unlike a closely related material, this mol­ecule readily forms large crystals by sublimation and by slow evaporation from di­chloro­methane. The maximum absorbance in the UV-Vis spectrum is at 533 nm. Emission was measured upon excitation at 533 nm with a fluorescence λ_max_ of 658 nm and cutoff of 900 nm.

## Abstract 2

The title compound crystallizes in space group *P*2_1_/*c* with four mol­ecules in the asymmetric unit.

## Graphical abstract

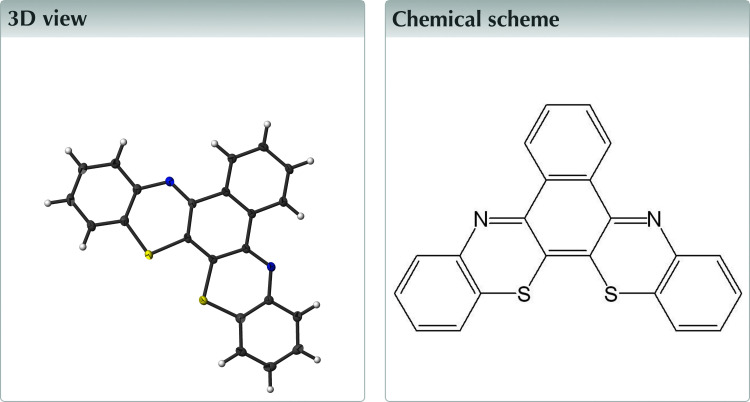

## Structure description

Fused heterocyclic aromatic compounds are of inter­est as an alternative to oligoacenes (Spangler *et al.*, 1989 ▸; McLean *et al.*, 1989 ▸, 1990 ▸; Pham *et al.*, 2008 ▸). Surprisingly, despite this intensely researched area, structural studies of these materials are scarce. Sulfur-containing fused heterocyclic compounds, such as pheno­thia­zine ladder polymers and oligomers are particularly inter­esting. Pheno­thia­zine systems can be obtained readily by reaction of halo-*p*-benzo­quinones and amino thio­phenols (Agarwal & Schaefer, 1980 ▸; Okafor *et al.*, 1988 ▸). The title compound was prepared as part of our work in crystalline organic semiconductors and was used in the construction of a single-crystal field effect transistor (Pham *et al.*, 2008 ▸).

**Figure 1:**
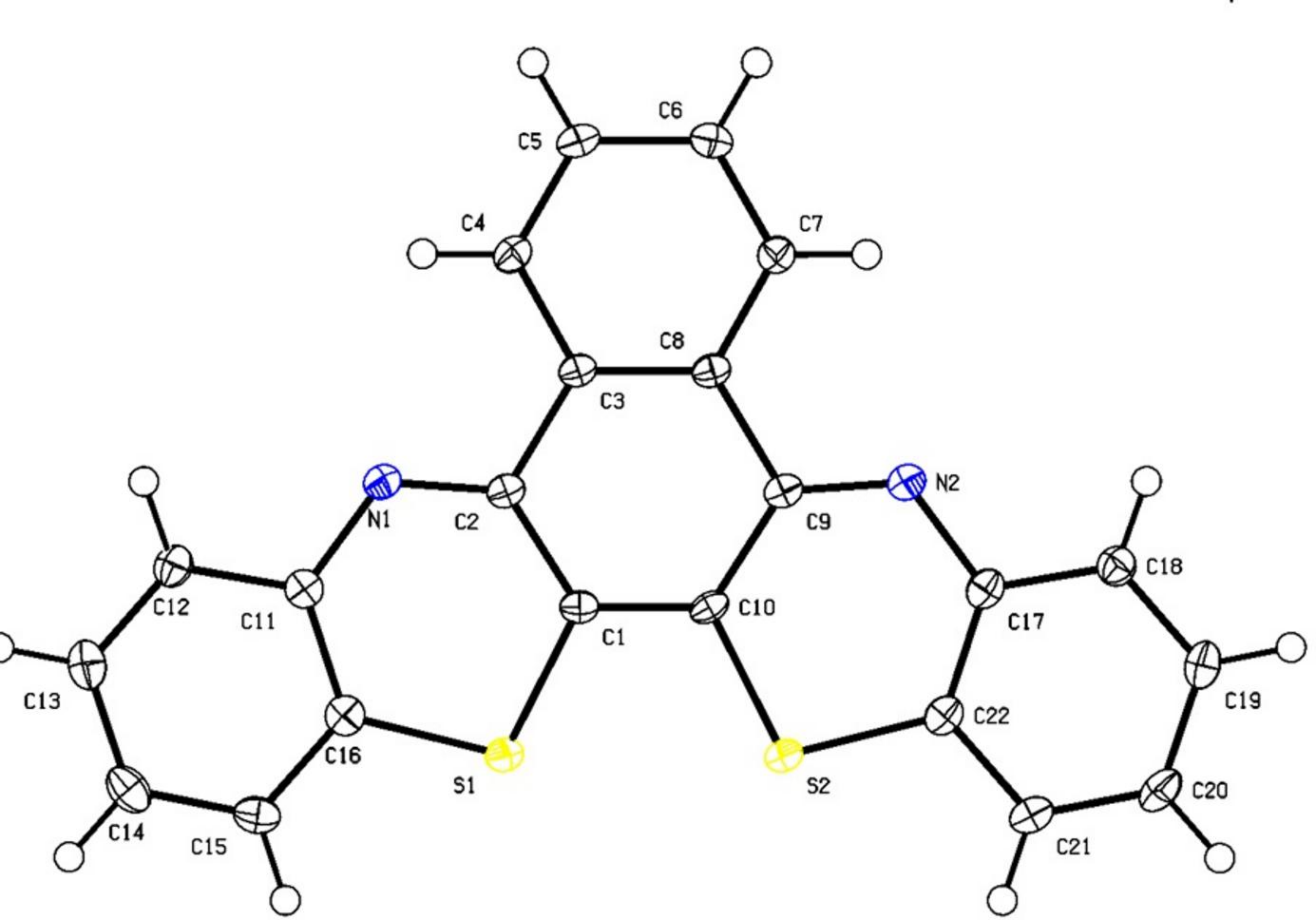
Structure of title compound with 50% probability ellipsoids.

**Figure 2:**
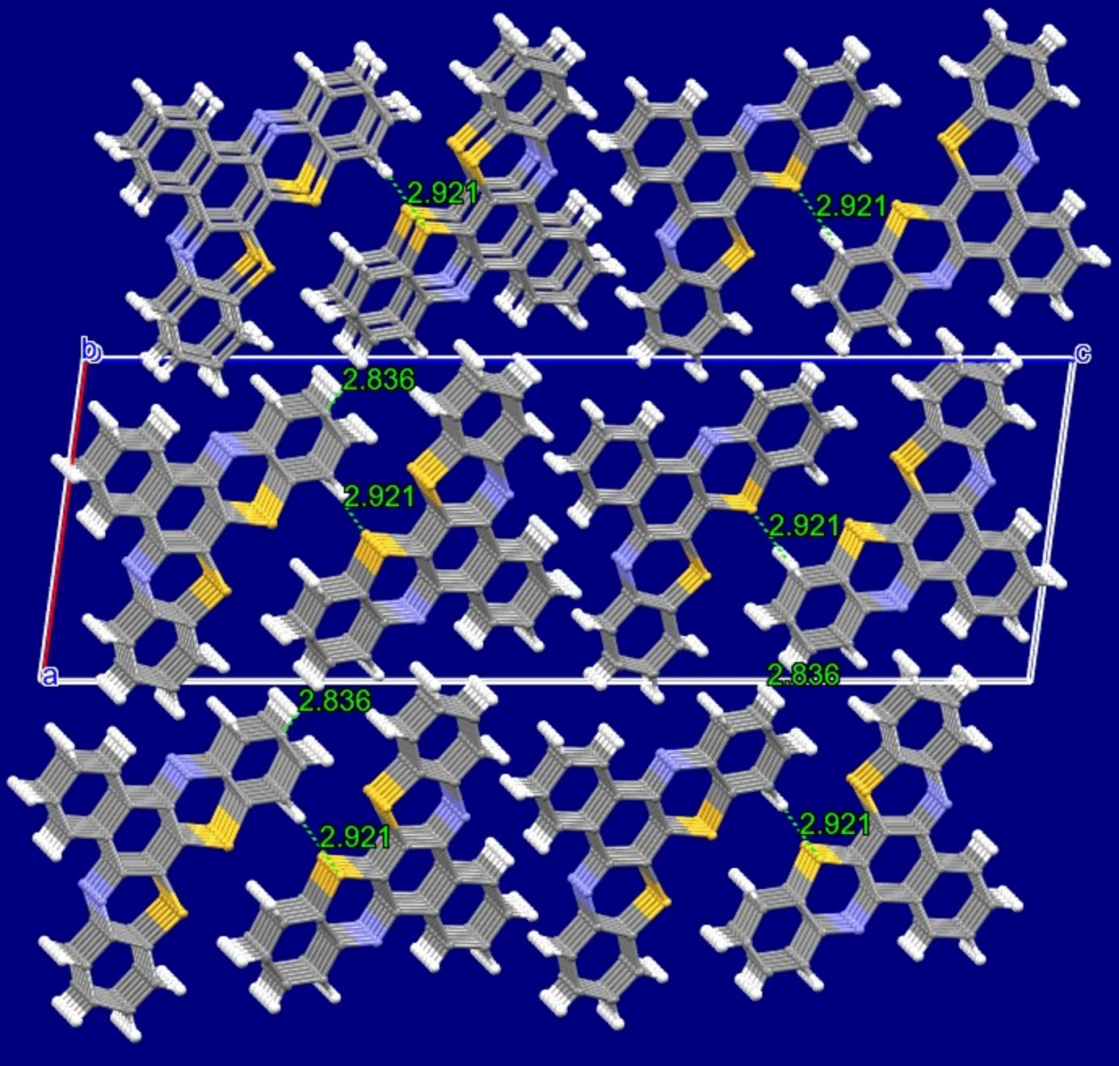
Packing of molecules governed by π-stacking and C—H⋯π contacts (H13⋯C13).

The molecule is quasi-planar (Fig. 1 ▸) with a dihedral angle between the C11–C16 and C17–C22 phenyl rings on the periphery of the mol­ecule of 1.73 (19)°. Individual mol­ecules stack along the *b* axis with π–π distances of 3.438 (3) Å between symmetry-related C3–C8 rings. The shortest inter­actions are H15⋯S1(1 − *x*, − 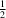 + *y*, 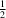 − *z*) = 2.92 Å and C—H⋯π [H13⋯C13(2 − *x*, 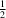 + *z*, 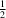 − *z*)] of 2.84 Å. The packing of molecules is shown in Fig. 2 ▸.

A survey of the Cambridge Structural Database (Groom *et al.*, 2016 ▸) on March 28, 2024 revealed no hits for this compound or any closely related structures.

**Figure 3:**
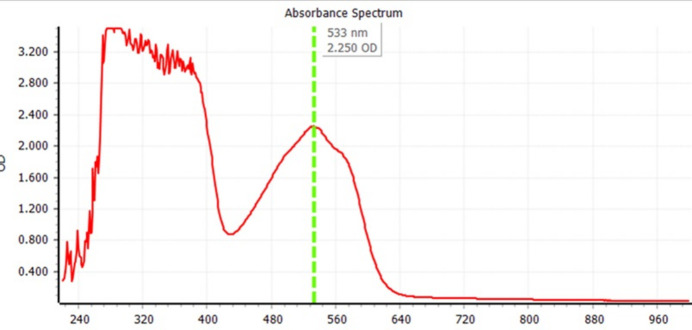
Uv–vis spectrum of the title compound in DMF.

This class of compounds have unique optical and electrical properties (Spangler *et al.*, 1989 ▸; Pham *et al.*, 2008 ▸; McLean *et al.*, 1990 ▸). The uv–vis spectra and florescence spectra are shown in Figs. 3 ▸ and 4 ▸. We note that the fluorescence cutoff spectra extend to 900 nm, which might be of inter­est for non-linear optical and biological applications.

## Synthesis and crystallization

The title compound was prepared followed published procedures (Feister *et al.*, 2023 ▸; Okafor, 1988 ▸). In a typical experiment, 2,3-di­chloro-1,4-naphtho­quinone (1 mmol, 1.0693 g) was dissolved in 10 ml DMF. 2-Amino­thio­phenol (2 mmol, 1 ml) was added to the solution. The reaction was then left stirring for 10 h, and the product was vacuum filtered. The product was then dried for 10 h in a vacuum oven, and then recrystallized from a di­chloro­methane solution, resulting in a dark-purple solid (0.7974 g, yield 46%), m.p. 280°C. Suitable crystals were grown either by sublimation or by slow evaporation from di­chloro­methane.

## Refinement

Crystal data, data collection and structure refinement details are summarized in Table 1 ▸.

## Supplementary Material

Crystal structure: contains datablock(s) I. DOI: 10.1107/S2414314624003572/oi4001sup1.cif

Structure factors: contains datablock(s) I. DOI: 10.1107/S2414314624003572/oi4001Isup5.hkl

CCDC reference: 2349720

Additional supporting information:  crystallographic information; 3D view; checkCIF report

## Figures and Tables

**Figure 4:**
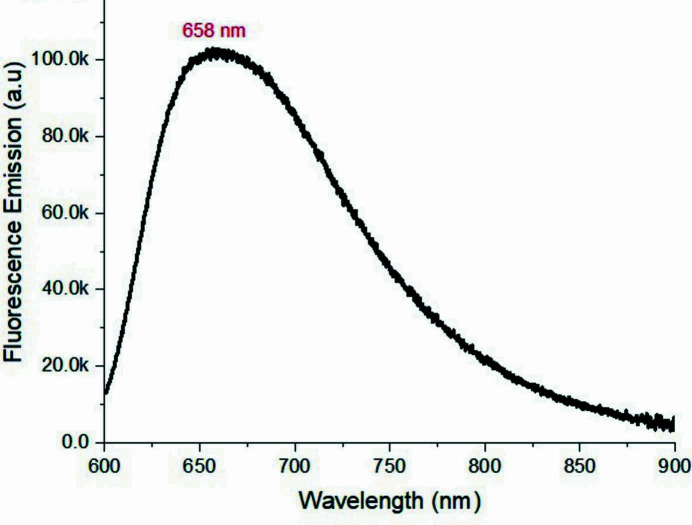
Emission spectrum of the title compound excited at 533 nm in DMF.

**Table 1:** Experimental details

Crystal data	
Chemical formula	C_22_H_12_N_2_S_2_
*M*_r_	368.46
Crystal system, space group	Monoclinic, *P*2_1_/*c*
Temperature (K)	100
*a*, *b*, *c* (Å)	11.7365 (5), 3.8149 (2), 35.7260 (15)
β (°)	97.669 (2)
*V* (Å^3^)	1585.27 (13)
*Z*	4
Radiation type	Mo *K*α
μ (mm^−1^)	0.34
Crystal size (mm)	0.25 × 0.06 × 0.01

Data collection	
Diffractometer	Bruker PHOTON-III CPAD
Absorption correction	Multi-scan (*SADABS*; Krause *et al.*, 2015 ▸)
*T*_min_, *T*_max_	0.642, 0.746
No. of measured, independent and observed [*I* > 2σ(*I*)] reflections	13015, 3927, 3341
*R*_int_	0.035
(sin θ/λ)_max_ (Å^−1^)	0.668

Refinement	
*R*[*F* ^2^ > 2σ(*F* ^2^)], *wR*(*F* ^2^), *S*	0.047, 0.116, 1.06
No. of reflections	3927
No. of parameters	235
H-atom treatment	H-atom parameters constrained
Δρ_max_, Δρ_min_ (e Å^−3^)	0.52, −0.56

Computer programs: *APEX4* and *SAINT* (Bruker, 2014 ▸), *SHELXT2018/2* (Sheldrick, 2015*a* ▸), *SHELXL2019/2* (Sheldrick, 2015*b* ▸) and *SHELXTL* (Sheldrick, 2008 ▸).

## full crystallographic data

### Crystal data

**Table:**

C_22_H_12_N_2_S_2_	*F*(000) = 760
*M**_r_* = 368.46	*D*_x_ = 1.544 Mg m^−^^3^
Monoclinic, *P*2_1_/*c*	Mo *K*α radiation, λ = 0.71073 Å
*a* = 11.7365 (5) Å	Cell parameters from 2950 reflections
*b* = 3.8149 (2) Å	θ = 2.3–28.3°
*c* = 35.7260 (15) Å	µ = 0.34 mm^−^^1^
β = 97.669 (2)°	*T* = 100 K
*V* = 1585.27 (13) Å^3^	Plate, brown
*Z* = 4	0.25 × 0.06 × 0.01 mm

### Data collection

**Table:**

Bruker PHOTON-III CPAD diffractometer	3341 reflections with *I* > 2σ(*I*)
Radiation source: micro-focus	*R*_int_ = 0.035
φ and ω scans	θ_max_ = 28.3°, θ_min_ = 2.0°
Absorption correction: multi-scan (SADABS; Krause *et al.*, 2015)	*h* = −15→15
*T*_min_ = 0.642, *T*_max_ = 0.746	*k* = −3→5
13015 measured reflections	*l* = −47→43
3927 independent reflections	

### Refinement

**Table:**

Refinement on *F*^2^	0 restraints
Least-squares matrix: full	Hydrogen site location: inferred from neighbouring sites
*R*[*F*^2^ > 2σ(*F*^2^)] = 0.047	H-atom parameters constrained
*wR*(*F*^2^) = 0.116	*w* = 1/[σ^2^(*F*_o_^2^) + (0.0466*P*)^2^ + 2.0952*P*] where *P* = (*F*_o_^2^ + 2*F*_c_^2^)/3
*S* = 1.06	(Δ/σ)_max_ = 0.001
3927 reflections	Δρ_max_ = 0.52 e Å^−^^3^
235 parameters	Δρ_min_ = −0.56 e Å^−^^3^

### Special details

**Table:**

Experimental. Prof. M. Bader/ Prof. D. Alvacado / A. Bradley
Geometry. All esds (except the esd in the dihedral angle between two l.s. planes) are estimated using the full covariance matrix. The cell esds are taken into account individually in the estimation of esds in distances, angles and torsion angles; correlations between esds in cell parameters are only used when they are defined by crystal symmetry. An approximate (isotropic) treatment of cell esds is used for estimating esds involving l.s. planes.

### Fractional atomic coordinates and isotropic or equivalent isotropic displacement parameters (Å^2^)

**Table:**

	*x*	*y*	*z*	*U*_iso_*/*U*_eq_	
S1	0.54183 (4)	0.21127 (14)	0.30740 (2)	0.01608 (13)	
S2	0.33242 (4)	0.20563 (14)	0.34472 (2)	0.01608 (13)	
C1	0.55313 (16)	0.3928 (5)	0.35287 (5)	0.0133 (4)	
C2	0.66054 (16)	0.5414 (5)	0.37063 (5)	0.0136 (4)	
C3	0.66375 (16)	0.6660 (5)	0.40993 (5)	0.0130 (4)	
C4	0.76638 (17)	0.7994 (6)	0.42939 (6)	0.0161 (4)	
H4	0.833194	0.811698	0.417086	0.019*	
C5	0.77115 (17)	0.9132 (6)	0.46631 (6)	0.0173 (4)	
H5	0.841216	1.001428	0.479273	0.021*	
C6	0.67283 (17)	0.8986 (6)	0.48468 (6)	0.0171 (4)	
H6	0.676060	0.977146	0.510033	0.021*	
C7	0.57106 (17)	0.7691 (5)	0.46564 (6)	0.0158 (4)	
H7	0.504467	0.759988	0.478081	0.019*	
C8	0.56486 (16)	0.6513 (5)	0.42822 (5)	0.0139 (4)	
C9	0.45459 (16)	0.5176 (5)	0.40854 (5)	0.0136 (4)	
C10	0.45630 (16)	0.3844 (5)	0.37040 (5)	0.0131 (4)	
N1	0.75471 (14)	0.5822 (5)	0.35518 (5)	0.0152 (3)	
C11	0.76197 (17)	0.4805 (5)	0.31800 (6)	0.0149 (4)	
C12	0.86667 (17)	0.5499 (6)	0.30445 (6)	0.0174 (4)	
H12	0.926848	0.662186	0.320482	0.021*	
C13	0.88363 (19)	0.4577 (6)	0.26821 (6)	0.0204 (4)	
H13	0.955212	0.505662	0.259611	0.025*	
C14	0.79652 (19)	0.2954 (6)	0.24432 (6)	0.0215 (5)	
H14	0.808693	0.231235	0.219467	0.026*	
C15	0.69185 (19)	0.2267 (6)	0.25667 (6)	0.0186 (4)	
H15	0.632088	0.117180	0.240201	0.022*	
C16	0.67390 (17)	0.3185 (5)	0.29338 (6)	0.0158 (4)	
N2	0.36590 (14)	0.5329 (5)	0.42687 (5)	0.0157 (4)	
C17	0.25691 (17)	0.4184 (6)	0.41125 (6)	0.0160 (4)	
C18	0.16828 (17)	0.4541 (6)	0.43387 (6)	0.0178 (4)	
H18	0.185008	0.553665	0.458358	0.021*	
C19	0.05728 (17)	0.3472 (6)	0.42117 (6)	0.0197 (4)	
H19	−0.001115	0.371277	0.437018	0.024*	
C20	0.03085 (18)	0.2041 (6)	0.38515 (6)	0.0210 (4)	
H20	−0.045527	0.132118	0.376360	0.025*	
C21	0.11667 (17)	0.1672 (6)	0.36219 (6)	0.0182 (4)	
H21	0.098693	0.071365	0.337562	0.022*	
C22	0.22938 (17)	0.2703 (5)	0.37509 (6)	0.0155 (4)	

### Atomic displacement parameters (Å^2^)

**Table:**

	*U*^11^	*U*^22^	*U*^33^	*U*^12^	*U*^13^	*U*^23^
S1	0.0154 (2)	0.0172 (3)	0.0152 (2)	−0.0022 (2)	0.00051 (17)	−0.00166 (19)
S2	0.0136 (2)	0.0180 (3)	0.0160 (2)	−0.0023 (2)	−0.00017 (17)	−0.00226 (19)
C1	0.0148 (9)	0.0119 (9)	0.0125 (9)	0.0016 (8)	−0.0013 (7)	0.0015 (7)
C2	0.0138 (9)	0.0115 (9)	0.0147 (9)	0.0007 (8)	−0.0007 (7)	0.0013 (7)
C3	0.0130 (8)	0.0111 (9)	0.0140 (9)	0.0009 (7)	−0.0012 (7)	0.0011 (7)
C4	0.0131 (8)	0.0160 (10)	0.0187 (10)	0.0001 (8)	0.0001 (7)	−0.0001 (8)
C5	0.0158 (9)	0.0164 (10)	0.0184 (10)	−0.0021 (8)	−0.0028 (7)	−0.0016 (8)
C6	0.0196 (10)	0.0165 (10)	0.0145 (9)	0.0015 (8)	−0.0003 (7)	−0.0014 (8)
C7	0.0154 (9)	0.0156 (10)	0.0161 (9)	−0.0001 (8)	0.0015 (7)	0.0009 (8)
C8	0.0136 (9)	0.0123 (9)	0.0148 (9)	0.0013 (8)	−0.0012 (7)	0.0006 (7)
C9	0.0139 (9)	0.0110 (9)	0.0150 (9)	0.0000 (8)	−0.0011 (7)	0.0017 (7)
C10	0.0116 (8)	0.0104 (9)	0.0160 (9)	−0.0003 (7)	−0.0025 (7)	0.0014 (7)
N1	0.0137 (8)	0.0155 (8)	0.0160 (8)	−0.0002 (7)	0.0001 (6)	−0.0003 (7)
C11	0.0167 (9)	0.0118 (9)	0.0163 (9)	0.0019 (8)	0.0018 (7)	0.0019 (7)
C12	0.0173 (9)	0.0137 (10)	0.0215 (10)	−0.0024 (8)	0.0035 (8)	0.0015 (8)
C13	0.0223 (10)	0.0167 (10)	0.0239 (11)	0.0020 (9)	0.0089 (8)	0.0024 (9)
C14	0.0299 (11)	0.0182 (10)	0.0175 (10)	0.0045 (9)	0.0068 (8)	0.0023 (8)
C15	0.0231 (10)	0.0153 (10)	0.0162 (10)	0.0016 (9)	−0.0013 (8)	−0.0002 (8)
C16	0.0185 (9)	0.0116 (9)	0.0172 (9)	0.0008 (8)	0.0020 (7)	0.0036 (8)
N2	0.0146 (8)	0.0158 (9)	0.0162 (8)	−0.0006 (7)	0.0006 (6)	0.0002 (7)
C17	0.0139 (9)	0.0148 (10)	0.0189 (10)	0.0014 (8)	0.0007 (7)	0.0031 (8)
C18	0.0168 (9)	0.0182 (10)	0.0182 (10)	0.0006 (8)	0.0022 (7)	0.0001 (8)
C19	0.0149 (9)	0.0196 (11)	0.0254 (11)	0.0016 (8)	0.0052 (8)	0.0032 (9)
C20	0.0130 (9)	0.0235 (11)	0.0252 (11)	−0.0022 (9)	−0.0021 (8)	0.0041 (9)
C21	0.0162 (9)	0.0168 (10)	0.0204 (10)	−0.0020 (8)	−0.0022 (7)	0.0001 (8)
C22	0.0139 (8)	0.0132 (10)	0.0187 (9)	0.0017 (8)	0.0002 (7)	0.0028 (8)

### Geometric parameters (Å, º)

**Table:**

S1—C16	1.740 (2)	C11—C12	1.405 (3)
S1—C1	1.755 (2)	C11—C16	1.407 (3)
S2—C22	1.747 (2)	C12—C13	1.382 (3)
S2—C10	1.751 (2)	C12—H12	0.9500
C1—C10	1.369 (3)	C13—C14	1.387 (3)
C1—C2	1.449 (3)	C13—H13	0.9500
C2—N1	1.309 (2)	C14—C15	1.385 (3)
C2—C3	1.478 (3)	C14—H14	0.9500
C3—C4	1.404 (3)	C15—C16	1.400 (3)
C3—C8	1.407 (3)	C15—H15	0.9500
C4—C5	1.382 (3)	N2—C17	1.396 (3)
C4—H4	0.9500	C17—C18	1.406 (3)
C5—C6	1.403 (3)	C17—C22	1.407 (3)
C5—H5	0.9500	C18—C19	1.383 (3)
C6—C7	1.384 (3)	C18—H18	0.9500
C6—H6	0.9500	C19—C20	1.394 (3)
C7—C8	1.403 (3)	C19—H19	0.9500
C7—H7	0.9500	C20—C21	1.389 (3)
C8—C9	1.479 (3)	C20—H20	0.9500
C9—N2	1.303 (2)	C21—C22	1.398 (3)
C9—C10	1.457 (3)	C21—H21	0.9500
N1—C11	1.398 (3)		
			
C16—S1—C1	102.32 (10)	C12—C11—C16	118.08 (18)
C22—S2—C10	102.41 (10)	C13—C12—C11	121.1 (2)
C10—C1—C2	122.38 (18)	C13—C12—H12	119.4
C10—C1—S1	116.71 (15)	C11—C12—H12	119.4
C2—C1—S1	120.91 (14)	C12—C13—C14	120.25 (19)
N1—C2—C1	126.58 (18)	C12—C13—H13	119.9
N1—C2—C3	116.52 (17)	C14—C13—H13	119.9
C1—C2—C3	116.89 (16)	C15—C14—C13	120.1 (2)
C4—C3—C8	119.40 (18)	C15—C14—H14	120.0
C4—C3—C2	119.92 (17)	C13—C14—H14	120.0
C8—C3—C2	120.68 (17)	C14—C15—C16	120.1 (2)
C5—C4—C3	120.62 (18)	C14—C15—H15	119.9
C5—C4—H4	119.7	C16—C15—H15	119.9
C3—C4—H4	119.7	C15—C16—C11	120.35 (19)
C4—C5—C6	120.19 (19)	C15—C16—S1	117.50 (16)
C4—C5—H5	119.9	C11—C16—S1	122.11 (15)
C6—C5—H5	119.9	C9—N2—C17	122.41 (17)
C7—C6—C5	119.59 (19)	N2—C17—C18	116.65 (18)
C7—C6—H6	120.2	N2—C17—C22	125.35 (18)
C5—C6—H6	120.2	C18—C17—C22	118.00 (18)
C6—C7—C8	120.95 (18)	C19—C18—C17	121.3 (2)
C6—C7—H7	119.5	C19—C18—H18	119.3
C8—C7—H7	119.5	C17—C18—H18	119.3
C7—C8—C3	119.24 (18)	C18—C19—C20	120.15 (19)
C7—C8—C9	119.63 (17)	C18—C19—H19	119.9
C3—C8—C9	121.12 (17)	C20—C19—H19	119.9
N2—C9—C10	126.69 (18)	C21—C20—C19	119.70 (19)
N2—C9—C8	116.92 (17)	C21—C20—H20	120.1
C10—C9—C8	116.40 (16)	C19—C20—H20	120.1
C1—C10—C9	122.38 (17)	C20—C21—C22	120.4 (2)
C1—C10—S2	116.83 (15)	C20—C21—H21	119.8
C9—C10—S2	120.79 (14)	C22—C21—H21	119.8
C2—N1—C11	122.00 (17)	C21—C22—C17	120.46 (18)
N1—C11—C12	116.25 (18)	C21—C22—S2	117.29 (16)
N1—C11—C16	125.67 (18)	C17—C22—S2	122.25 (15)
			
C16—S1—C1—C10	173.32 (16)	C1—C2—N1—C11	−0.2 (3)
C16—S1—C1—C2	−7.01 (19)	C3—C2—N1—C11	−178.92 (17)
C10—C1—C2—N1	−175.1 (2)	C2—N1—C11—C12	177.87 (19)
S1—C1—C2—N1	5.3 (3)	C2—N1—C11—C16	−1.7 (3)
C10—C1—C2—C3	3.7 (3)	N1—C11—C12—C13	179.44 (19)
S1—C1—C2—C3	−175.95 (14)	C16—C11—C12—C13	−0.9 (3)
N1—C2—C3—C4	−3.8 (3)	C11—C12—C13—C14	0.4 (3)
C1—C2—C3—C4	177.34 (18)	C12—C13—C14—C15	0.4 (3)
N1—C2—C3—C8	176.48 (19)	C13—C14—C15—C16	−0.5 (3)
C1—C2—C3—C8	−2.4 (3)	C14—C15—C16—C11	−0.1 (3)
C8—C3—C4—C5	0.5 (3)	C14—C15—C16—S1	−177.97 (17)
C2—C3—C4—C5	−179.23 (19)	N1—C11—C16—C15	−179.62 (19)
C3—C4—C5—C6	−0.5 (3)	C12—C11—C16—C15	0.8 (3)
C4—C5—C6—C7	0.1 (3)	N1—C11—C16—S1	−1.9 (3)
C5—C6—C7—C8	0.2 (3)	C12—C11—C16—S1	178.56 (16)
C6—C7—C8—C3	−0.1 (3)	C1—S1—C16—C15	−176.64 (16)
C6—C7—C8—C9	−179.42 (19)	C1—S1—C16—C11	5.5 (2)
C4—C3—C8—C7	−0.2 (3)	C10—C9—N2—C17	−0.2 (3)
C2—C3—C8—C7	179.50 (18)	C8—C9—N2—C17	179.35 (18)
C4—C3—C8—C9	179.07 (19)	C9—N2—C17—C18	−179.1 (2)
C2—C3—C8—C9	−1.2 (3)	C9—N2—C17—C22	1.2 (3)
C7—C8—C9—N2	3.3 (3)	N2—C17—C18—C19	−179.7 (2)
C3—C8—C9—N2	−176.02 (19)	C22—C17—C18—C19	0.1 (3)
C7—C8—C9—C10	−177.15 (18)	C17—C18—C19—C20	−0.7 (3)
C3—C8—C9—C10	3.5 (3)	C18—C19—C20—C21	0.4 (3)
C2—C1—C10—C9	−1.3 (3)	C19—C20—C21—C22	0.5 (3)
S1—C1—C10—C9	178.35 (15)	C20—C21—C22—C17	−1.1 (3)
C2—C1—C10—S2	178.49 (15)	C20—C21—C22—S2	178.64 (17)
S1—C1—C10—S2	−1.8 (2)	N2—C17—C22—C21	−179.5 (2)
N2—C9—C10—C1	177.2 (2)	C18—C17—C22—C21	0.8 (3)
C8—C9—C10—C1	−2.3 (3)	N2—C17—C22—S2	0.8 (3)
N2—C9—C10—S2	−2.6 (3)	C18—C17—C22—S2	−178.90 (16)
C8—C9—C10—S2	177.87 (14)	C10—S2—C22—C21	177.42 (17)
C22—S2—C10—C1	−176.21 (16)	C10—S2—C22—C17	−2.8 (2)
C22—S2—C10—C9	3.60 (18)		

## References

[bb1] Agarwal, N. L. & Schaefer, W. (1980). *J. Org. Chem.* **45**, 2155–2161.

[bb3] Bruker (2014). *APEX2* and *SAINT*. Bruker AXS Inc., Madison, Wisconsin, USA.

[bb4] Feister, C., Pham, P.-T. T. & Bradley, A. (2023). *MRS Advances*, pp. 889–893. https://doi.org/10.1557/s43580-023-00609-y

[bb5] Groom, C. R., Bruno, I. J., Lightfoot, M. P. & Ward, S. C. (2016). *Acta Cryst.* B**72**, 171–179. 10.1107/S2052520616003954 PMC4822653 27048719

[bb6] Krause, L., Herbst-Irmer, R., Sheldrick, G. M. & Stalke, D. (2015). *J. Appl. Cryst.* **48**, 3–10. 10.1107/S1600576714022985 PMC4453166 26089746

[bb7] McLean, M. R., Bader, M., Dalton, L. R., Devine, R. S. & Steier, W. H. (1990). *J. Phys. Chem.* **94**, 4386–4387.

[bb8] McLean, M. R., Bader, M., Dalton, L. R., Devine, L. R. S. & Steier, W. H. (1989). *MRS Online Proceedings Library*, **173**, 563–566. https://doi.org/10.1557/PROC-173-563

[bb9] Okafor, C. O. (1988). *Tetrahedron*, **44**, 1187–1194.

[bb11] Pham, P.-T., Xia, Y., Frisbie, C. D. & Bader, M. (2008). *J. Phys. Chem. C*, **112**, 7968–7971.

[bb12] Sheldrick, G. M. (2008). *Acta Cryst.* A**64**, 112–122. 10.1107/S0108767307043930 18156677

[bb13] Sheldrick, G. M. (2015*a*). *Acta Cryst.* A**71**, 3–8.

[bb14] Sheldrick, G. M. (2015*b*). *Acta Cryst.* C**71**, 3–8.

[bb15] Spangler, C. W., Havelka, K., Bader, M. M., McLean, M. R. & Dalton, L. R. (1989). *Proc. SPIE*, **1147**, 149.

